# Environmental impact assessment of leachate from mining tailings using electrical resistivity imaging

**DOI:** 10.1038/s41598-025-08030-1

**Published:** 2025-07-02

**Authors:** Mosaad Ali Hussein Ali, Wei Qian, Ragab Rabeiy, Hussein A. Saleem, Ahmed S. Mohamed, Abdullahi Uwaisu Muhammad, Ali Shebl

**Affiliations:** 1 Mining and Metallurgical Engineering Department, Assiut University, Assiut, 71515 Egypt; 2 School of Earth Sciences and Engineering, Hohai University, Nanjing, 211100 China; 3 Department of Civil Engineering, College of Engineering, Qassim University, Buraydah, 51452 Saudi Arabia; 4 Mining Engineering Department, King Abdulaziz University, Jeddah, 21589 Saudi Arabia; 5 Mining and Petroleum Department, Faculty of Engineering-Qena, Al-Azhar University, Qena, 83513 Egypt; 6 Department of Computer Science, Federal University Dutse, Dutse, 7156, Nigeria; 7 Department of Geology, Tanta University, Tanta, 31527 Egypt; 8 Department of Mineralogy and Geology, University of Debrecen, Debrecen, 4032 Hungary

**Keywords:** Mining tailings, Leachate pre-assessment, Electrical resistivity imaging, Legacy dump, Environmental management, Modelling, Environmental sciences, Hydrology, Solid Earth sciences, Space physics

## Abstract

The environmental difficulties from mining tailings arise mainly from legacy dump sites because these residues spread pollution through surrounding areas. Effective environmental management requires a comprehensive pre-assessment. An ERI, electrical resistivity imaging, system serves as the analytical tool to create models for leachate assessment prior to its measurement in abandoned mining tailing storage sites. A total of 16 2D ERI profiles produced both 2D and 3D models that monitored the El Mochito mine waste site in Honduras. Different geoelectric zones were identified in the electrical resistivity models of this site with high resistivity values ranging between 60 and 100 Ω m in the surface layer while the middle layer exhibited moderate resistivity between 30 and 60 Ω m and the lowest resistivity of 1–30 Ω m was observed in the active leaching zone that contained conductive materials and mineral-rich leachate. The 3D hydrogeological models provided clear visibility of leachate areas and flow paths. The leachate migration showed uniform movement towards the northern direction until it reached the southern region where concentrations decreased. Another level of spatial understanding and depth information on resistivity distribution was obtained from 3D ERI models. The complete assessment objectives of the research form the basis for future investigations while demonstrating the importance of integrating geochemical measurements. The study emphasizes the need for ERI to examine complicated mining tailings yet requests deeper scientific investigation to create effective environmental management techniques and remediation practices.

## Introduction

Tailings from mining operations contain large volumes of pulverized rock fragments together with process waste products^1–4^. The disposal practice of tailings occurs in designated containment areas or dumps causing critical environmental risks. One critical matter of concern involves leachate spreading through nearby locations because this substance contains dissolved minerals together with other materials. The difficulties associated with legacy dumps increase because they were created using older disposal approaches that fall below present-day requirements^5,6^. The study of leachate formation together with its migration behavior and containment procedures within mining tailings dumps demands highest priority. The improper management of tailings leads to severe to catastrophic environmental damage according to sources^7,8^. The monitoring of tailings leachate spreading needs to be consistently performed for immediate cleanup work and forthcoming mining venture design purposes. Achieving mapping and monitoring of mining tailings requires an effective tool because the continuous control of their environmental impact remains crucial^3,9–14^.

The delineation and assessment of leachate migration effects from landfills on environments traditionally use standard testing methods like monitoring groundwater along with collecting surface water data and analyzing soil contents through monitoring wells installation around a pond/dump site^15–17^. Traditional methods deliver crucial hydrogeological and chemical information which requires long durations, extensive labor, and produces restricted small-scale results^18^. Invasive procedures involved in these methods cause damage to the subsurface environment while their inability to detect complete leachate migration becomes a challenge especially when operating in complex geological structures. A successful approach for environmental risk management related to mining tailings necessary includes detailed pre-assessments combined with ongoing monitoring.

Geophysical systems serve as standard tools for investigating groundwater pollution that occurs from hydrocarbon leaks and garbage disposal sites and salt water encroachment. These techniques have become standard globally for defining tailing deposits and their geological features along with detecting groundwater and protection layers^19,20^. The main techniques for analyzing tailing materials involve Electrical Resistivity Imaging (ERI) together with Self-Potential (SP) and Induced Polarization (IP) along with Ground Penetrating Radar (GPR). The international scientific community has utilized Seismic Refraction Tomography, SRT, to investigate how tailings and historical waste deposits spread out across different sites^16,21,22^. Most investigations concerning tailing dams concentrate on evaluating the dams’ characteristics yet the unregulated spread of tailings throughout the underground layers remains inadequately studied. The impact assessment procedure for leachate movement and contamination levels within tailings storage facilities (TSFs) represents one of the major challenges in many countries. For example, Swedish mining operations produce waste rock and considerable amounts of tailings which get stored in tailing impoundments after their generation. The Hammaslahti Cu–Zn mine in Eastern Finland benefits from terrestrial geophysical method applications through gravity measurements combined with seismic refraction and electromagnetic surveys and resistivity soundings to evaluate tailing impoundments and their underlying bedrock and soil perimeter structures according to^23^. ERI functions as one of the essential non-invasive geophysical techniques which serves both hydrogeological research requirements and the mapping needs of mine waste tailings^24–27^. The identification of groundwater mapping and its related contaminants depends heavily on ERI survey operations^28–35^. The essential drivers that affect ERI results include water saturation changes alongside temperature and ion content variations because ERI has become the standard technique for studying solute transport and groundwater mapping^36–40^. ERI successfully identifies paths through which leachate moves through mining tailings so researchers can monitor and characterize mining objects like tailings dams and abandoned underground excavation sites^41,42^. The ERI demonstrates exceptional value as a geophysical method which delivers essential subsurface information through its helping initial assessment of how leachate from mining tailings spreads throughout legacy dumps^43^.

The effectiveness of ERI technology in tailings leachate assessment has received little specificity despite successful applications for general groundwater contamination and landfill leachate migration. Research on leachate monitoring utilizing geophysical techniques has been focused on active tailings storage facilities yet studies on legacy dumps remain scarce because their hydrogeochemical behaviors become intricate through natural weathering and past disposal methods. Most available research depended on 2D resistivity models to monitor leachate movement but these models deliver minimal spatial insights about leachate movement. Research dedicated to combining 3D ERI models remains limited especially for areas with varying hydrological settings which affect leachate movement through seasonal floods and heavy precipitation. The El Mochito mine in north-western Honduras, which operates on sulfide ores, generates diverse forms of tailings comprising both solid and liquid components^42^. The mining process yields lead (Pb), zinc (Zn), and silver (Ag) as primary products, generating substantial quantities of waste^41,44^. Tailings storage facilities receive the liquid tailings after separating them from solid waste materials through a pipeline from the treatment plant. However, the tropical geographical context of El Mochito mine, characterized by frequent floods and heavy rainfall, poses unique challenges in tailings management^44^.

This study aims to develop hydrogeological simulation models through ERI to delineate leachate movement and contamination levels within the mining tailings of a particular dump at the El Mochito mine waste site. By integrating 2D and 3D ERI models, this approach seeks to:

- Delineate leachate pathways and identify contamination zones.
- Understand subsurface hydrogeological conditions, including variations in moisture content and saturation levels.
- Enhance predictive modeling by linking ERI data with hydrogeological simulations to forecast leachate dispersion over time.
- Support environmental management and remediation efforts by providing detailed subsurface information crucial for designing containment and treatment strategies.

While this approach represents an initial step in environmental impact assessment, it holds paramount importance in guiding informed decision-making for effective environmental management and remediation of mining tailings in legacy dumps. This research emphasizes the significance of advanced geophysical techniques like ERI in understanding and controlling tailings leachates, ultimately promoting environmental sustainability within the mining industry.

## Materials and methods

### Location and description

**Fig. 1:**
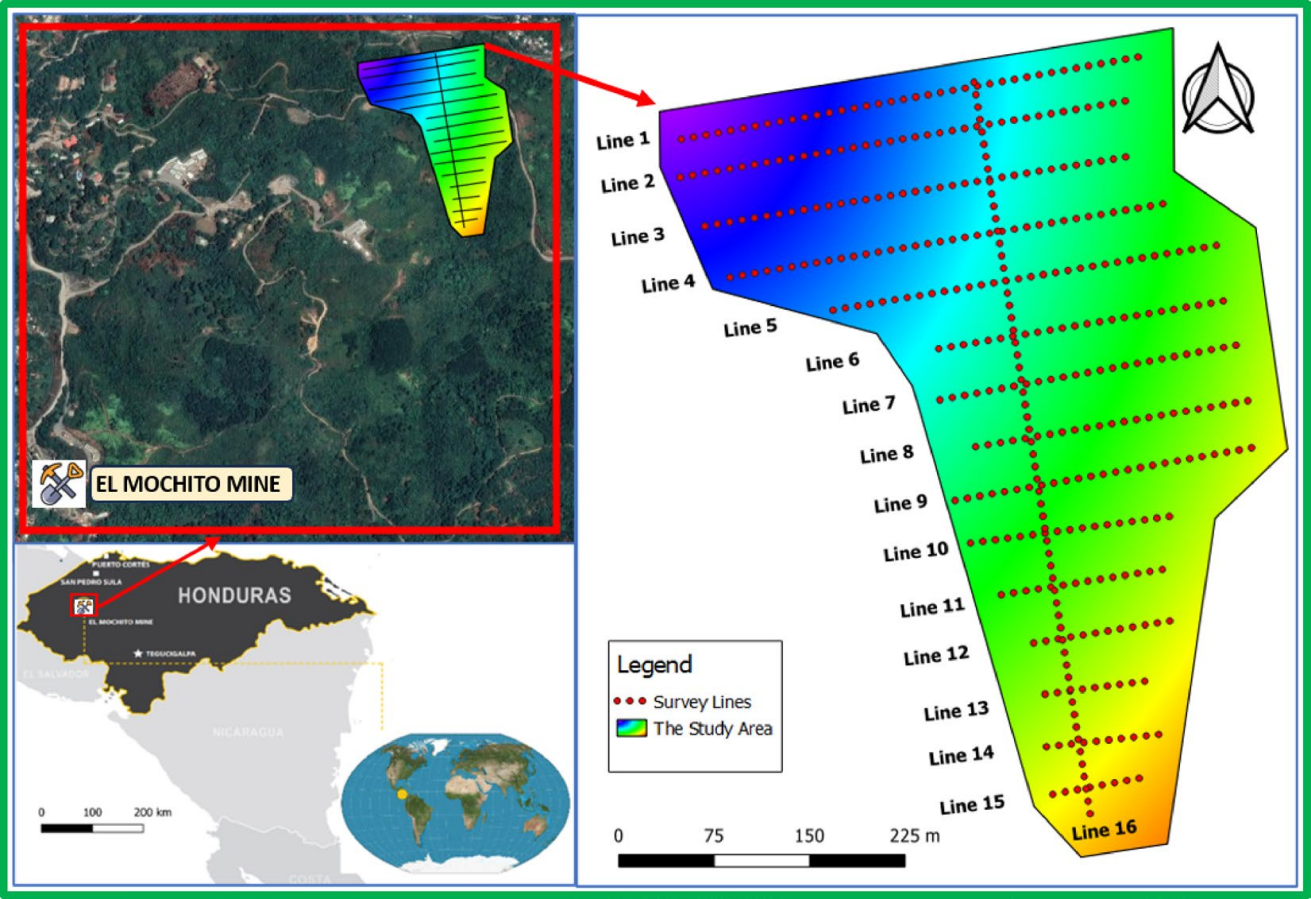
Location of the El Mochito Mine in Honduras (inset map) and a detailed view of the study area with the grid of survey lines. The color gradient within the study area likely represents a measured geophysical property (the satellite imagery and the grid of survey lines of the study area generated using QGIS Desktop 3.28.2, https://www.qgis.org).

El Mochito mine site occupies northwest Honduras within proximity to Las Vegas town with distances of 88 km southwest from San Pedro Sula and 220 km northwest from Tegucigalpa as displayed in Fig. 1. The survey site covers a range from − 88.0699, 14.8621 to − 88.0648, 14.8667 and runs under EPSG:4267-NAD27 coordinate reference.

**Fig. 2:**
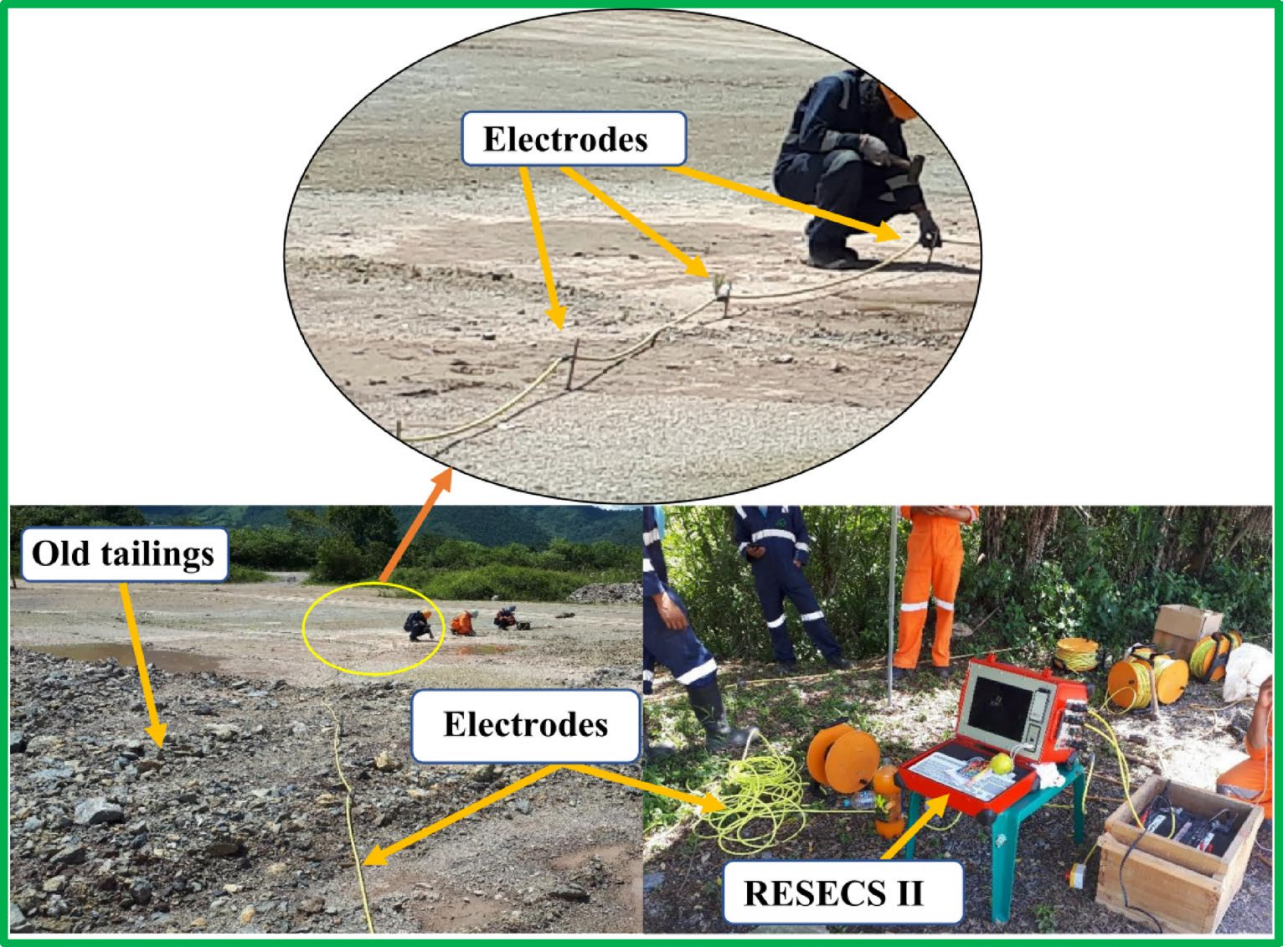
Field setup of the EarthProbe system, illustrating the electrode array deployed on old tailings and the connected RESECS II control unit with its associated accessories.

The El Mochito property functions as an underground mining operation for Pb–Zn–Ag ore that leads to the production of separate lead and zinc concentrates at its concentrator facility. Traditional differential sulfide flotation occurs at the El Mochito mine processing site which handles a daily throughput of approximately 2,250 t. The waste generation in flotation plants amounts to more than 300 tonnes per day. The treating plant routes excess waste material through an extensive 4.5-km pipelines before filtering out liquid matter to send solids to tailings-storage-facilities (TSF). Three TSFs located in El Bosque Soledad and Pozo Azul provide continuous operation for mining activities. The El-Bosque dump, as shown in Fig. 2, (the site on which this study is being conducted) is the earliest and holds around 5 million tons (Mt) of old mining tailings. This dump’s surface has been re-vegetated naturally after officially closing in 2018. There was an underground decant system and a settling pond at the toe of the dam in the TSF to filter the clean water from solid waste materials. Also, the Nyrstar mine administration constructed a 180 m long retaining wall down of the dam for preventing potential soil erosion because of weathering factors such as rainfalls and flooding the river of Quebrada Raices^44^. In addition to all these precautions, the company monitors from time to time the state of water seepage/tailings leachate through old tailing to ensure the safety of the environment. One of the ways of this monitoring is to detect and map tailings leachate using geophysical techniques to know the extent of penetration and spread of pollutants underground. Geophysical techniques such as ERI are well-established techniques for hydrogeological studies to monitor and map the extent of tailings leachates for both treatments and future planning considerations. Therefore, we used 2D ERI in this study for subsurface mapping of mine tailings’ leachates in El Bosque dump.

### Electrical resistivity imaging (ERI) technique

ERI stands as a non-invasive geophysical method which creates images while understanding geological subsurface properties of formations below ground^45^. The process of ERI consists of applying electrical currents into the soil to detect electrical or conductive properties of subterranean materials and measure the generated voltage distribution patterns. The spatial analysis of electrical resistivity values produces 2D or 3D subsurface images through ERI to provide relevant data for environmental analyses and geotechnical examinations as well as hydrogeological evaluations and mineral prospecting^46–48^. The ramification of ERI occurs through assessment of electrical resistivity values which manifests from material properties of electrical resistivity and its inverse property electrical conductivity. Electrical resistivity acts as a measure which indicates how much a material resists the movement of electrical currents through it. The measurement unit used to represent it is Ohm-meters. The resistivity measurements of insulating substances such as arid soil and stones and solid rock fall in the range of several hundred to several thousand Ohm-meters. Such materials function by limiting electrical current movements. Water metals and saline solutions display very low resistivity values that measure in fractions of Ohm-meters according to^49–51^. The materials enable electrical current to flow freely. The measured data consisting of electric current application spots along with their recorded voltage measurements allow the creation of a computational model representing subsurface resistivity. The data acquisition requires mathematical inversion algorithms to calculate resistivity distributions matching the recorded observations^52–55^.

### Equipment and data acquisition

To obtain a detailed subsurface characterization of the tailings, an Electrical Resistivity Imaging (ERI) survey was conducted using the EarthProbe high-resolution DCIP system, as shown in Fig. 2. This system can be configured for collecting high-resolution surface Induced Polarization (IP) data, vertical profiles (VP), and multi-borehole/surface-to-borehole tomographic images. For this study, data acquisition was performed using the high-resolution surface DC configuration. Only voltage and current were measured, allowing for the calculation of apparent resistivity. A summary of the survey system specifications is provided in Table 1.

**Table 1:** Specifications of the EarthProbe system.

Survey item	Specifications
Survey type	Direct current resistivity
Geophysical system	EarthProbe High Resolution surface and borehole DCIP system
Data type	Full-waveform, 256 ms on-time and 256 ms off-time, castle waveform
Survey configuration	Surface DCIP: Wenner Alpha array
Voltage input	The system uses 12 V DC, it has a transformer inside to convert 12 V into 24 V to 800 V
Electrode spacing	2.3 m

DC apparent resistivity data were collected along 16 surface profiles—15 parallel profiles oriented east–west and one perpendicular profile running north–south—spaced 40 m apart, with an electrode separation of 2.3 m, as illustrated in Fig. 1. The ERI data were acquired using the Wenner-alpha array, where stainless steel stakes served as both current electrodes (A–B) and potential electrodes (M–N). In this configuration, electrodes are equally spaced in the order A–M–N–B, with the “a-spacing” increasing incrementally for each reading. For this survey, the electrical current waveform was generated using a 256-ms (ms) square wave cycle, consisting of a 256 ms positive charge, 256 ms off, 256 ms negative charge, and another 256 ms off period.

### Data processing

**Fig. 3:**
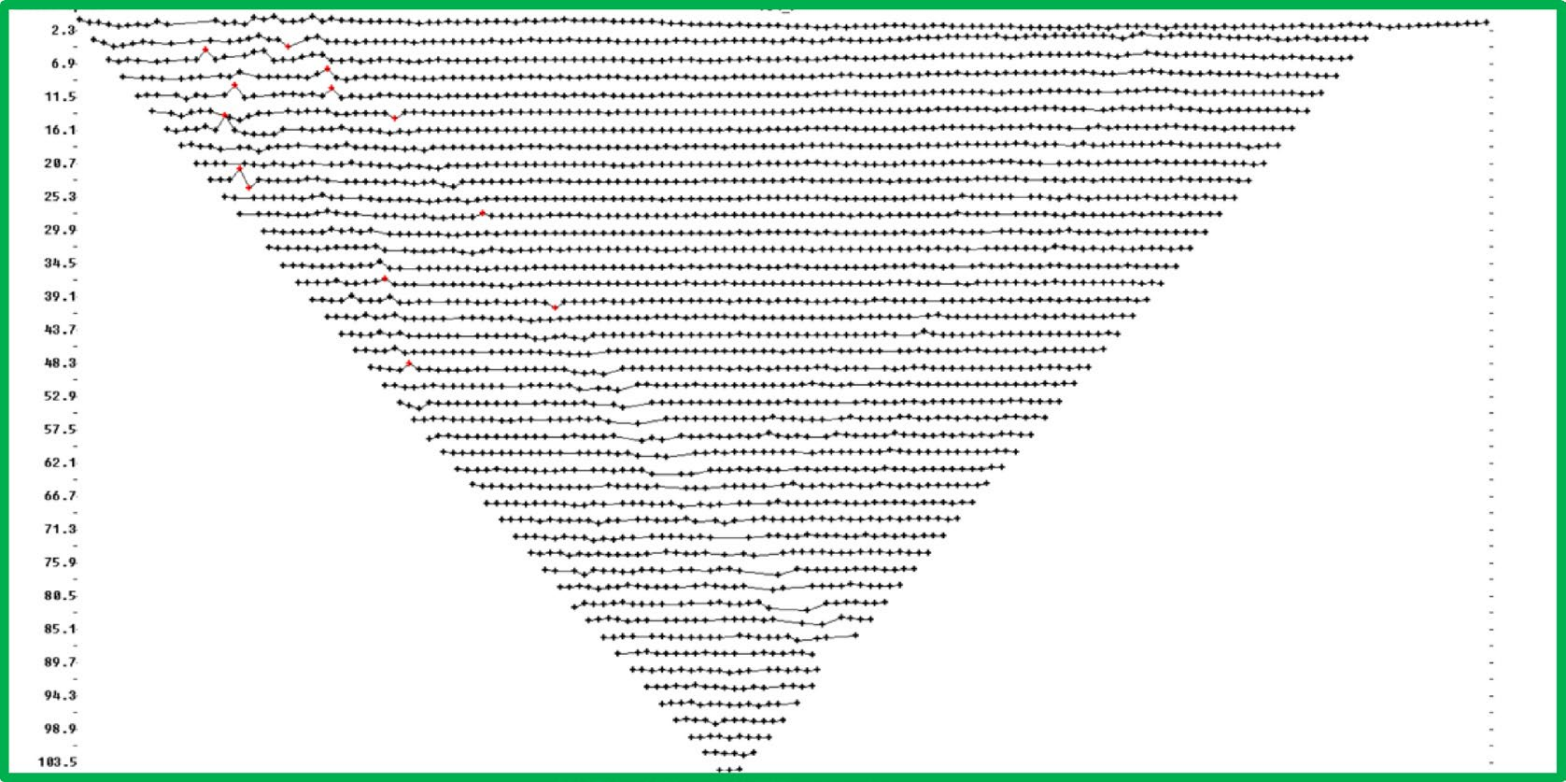
Example of a geophysical data set exhibiting several flawed data points, highlighted in red, which may require further processing or removal.

Data processing is a critical step in ERI surveys, as it involves transforming raw field measurements into meaningful subsurface resistivity images. The data processing and inversion steps used Res2DInv and Res3DInv software packages. The software tools used for ERI data processing inversion and interpretation belong to common packages described in multiple publications starting from reference numbers^56–58^. This section details step-by-step instructions regarding data processing through software packages focusing on methods to create trustworthy resistivity models from obtained ERI field measurements. Start the software as the first step and then import all ERI data points obtained from field survey operations. The first step involves quality control assessments to guarantee data precision. And if necessary boil out the points of interest then verify that none of the ERI data contain abnormal points or values which stem from measurement errors or noise. The main aim of this procedure is to remove data points with obviously wrong resistivity results. The incorrect data points stem from broken electrode relays or unsteady ground-electrode contact due to dry terrain or from damaged cables during moist conditions. The erroneous data points present in the ERI exhibit highly outlying apparent resistivity measurements compared to their neighboring measurements. It is most beneficial for the analysis to eliminate these ERI data points because their elimination prevents them from shaping the final model design^56–58^. Figure 3 shows a data set wherein a few red-marked data points represent the flawed measurements.

**Fig. 4:**
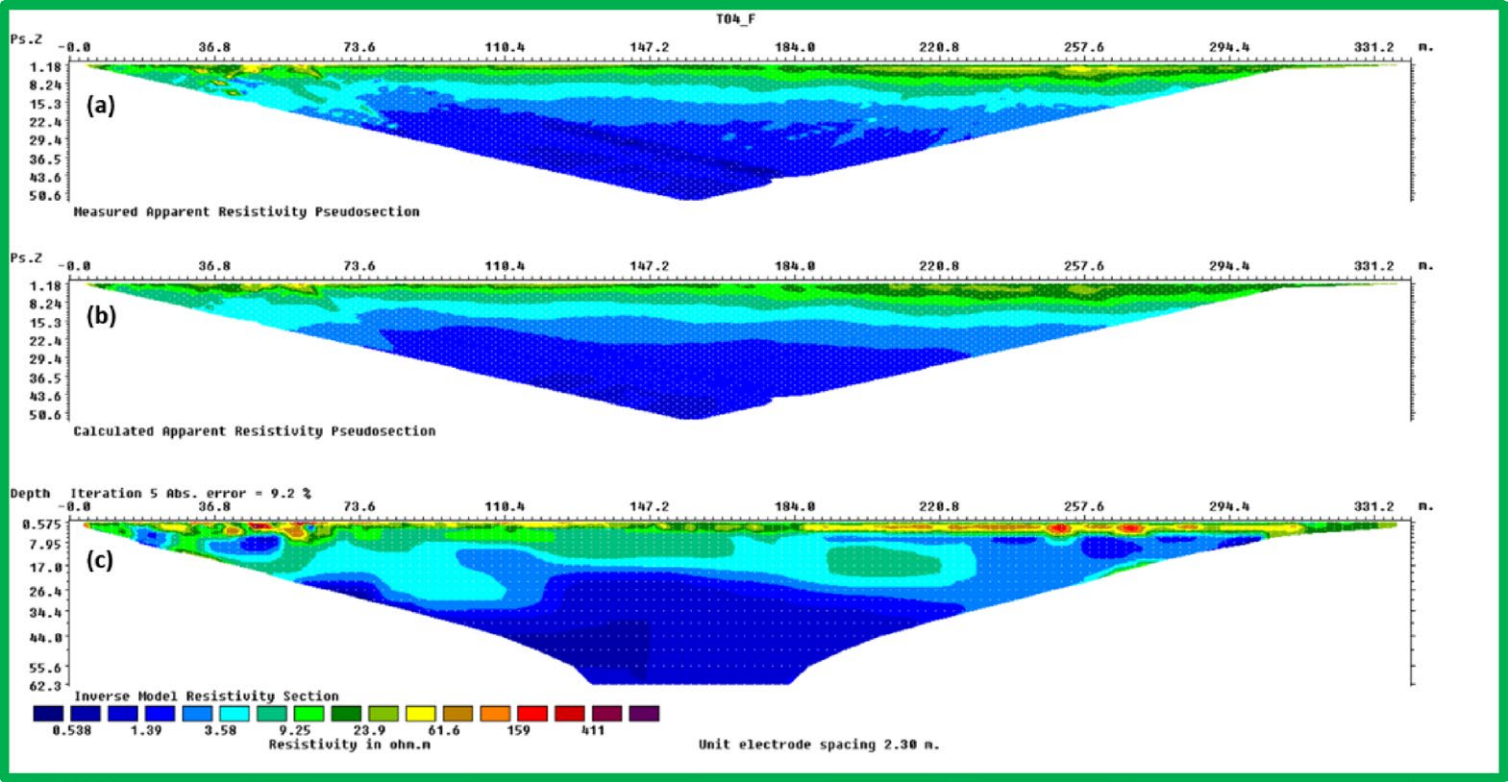
2D Electrical Resistivity Imaging (ERI) sections along a survey line: (**a**) Measured apparent resistivity pseudosection, displaying the raw data distribution; (**b**) Calculated apparent resistivity pseudosection, representing the model response after inversion; and (**c**) Inverse model resistivity section, showing the interpreted subsurface resistivity distribution in ohm-meters.

Forward modeling requires creating all required parameters for simulation work. The setup requires specification of survey dimensions along with electrode array types including Wenner Schlumberger or dipole–dipole and measurements distance and calculation of pseudosection (Fig. 4a). A forward model simulation provides expected apparent resistivity values that stem from initial resistivity models. Synthetic ERI data (Fig. 4b) is created through this process which will be assessed parallel to acquired ERI data during the inversion procedures.

**Fig. 5:**
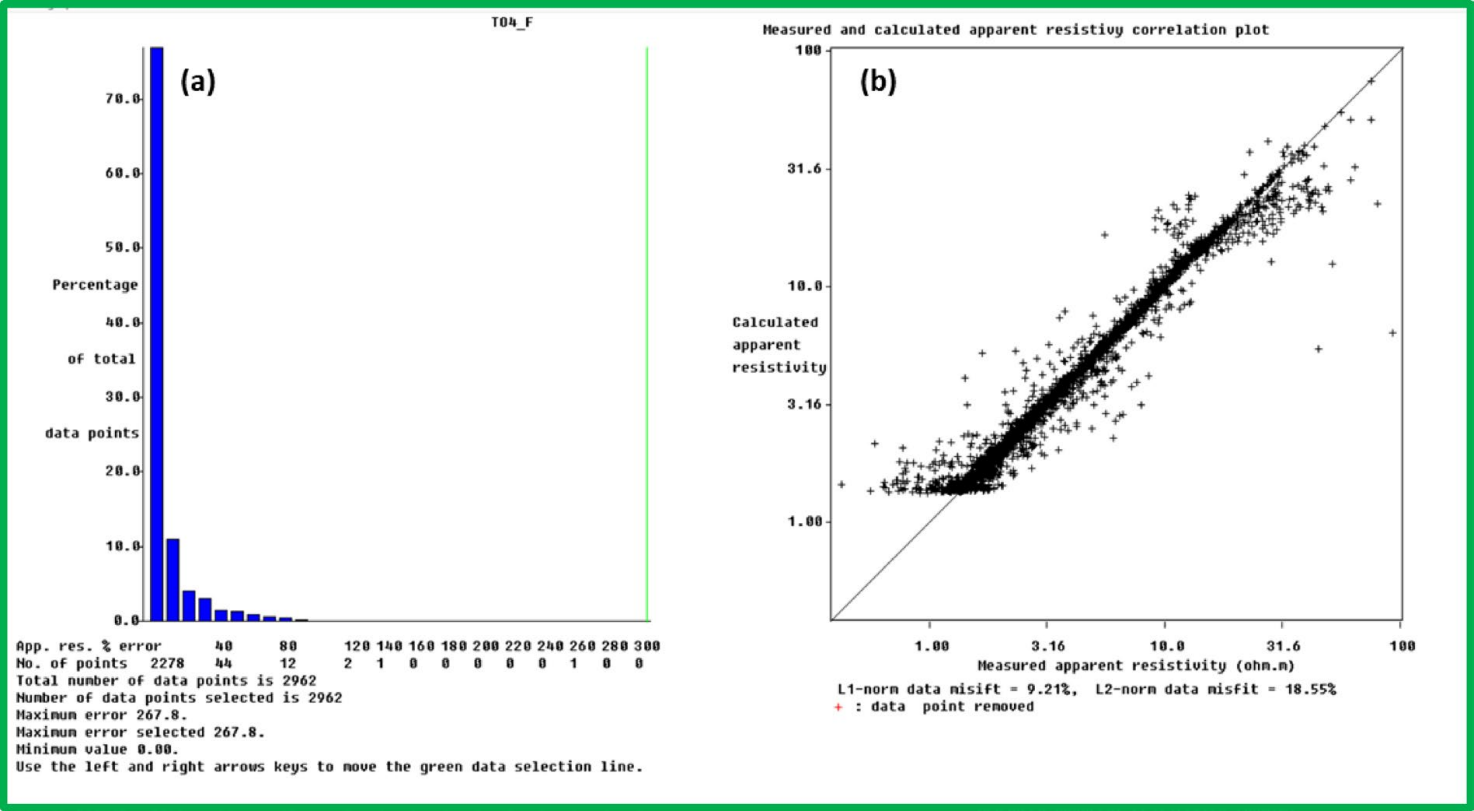
Assessment of inversion error for the 2D resistivity model before outlier removal: (**a**) Histogram displaying the distribution of absolute percentage errors between calculated and measured apparent resistivity values, indicating the overall data misfit; and (**b**) Scatter plot illustrating the correlation between measured and calculated apparent resistivity, with deviations from the ideal 1:1 line representing the misfit. The L1-norm and L2-norm data misfits are also provided.

The inversion technique modifies sub-surface resistivity distributions to achieve optimal fit with observed data through its operating framework (Fig. 4c). Users need to establish necessary inversion parameters which consist of selecting the inversion method between smoothness-constrained and Occam along with choosing damping factors and convergence rules. The chosen parameters determine how the inversion performs its regularization function as well as its ability to reach convergence. The process begins with choosing an appropriate starting resistivity model either from existing geological data or preliminary subsurface hypothesis. The inversion begins with this step so the software performs successive value adjustments in the model to decrease the measurement differences between synthetic and observed data (Fig. 5). The method continues until it reaches convergence state after performing a pre-defined number of iterations.

The system produces complete pictorial results and data set views of the ultimate resistivity model. The reports need to contain geological interpretations together with anomaly identifications combined with suggestions resulting from the findings. You should employ visualization tools within Res2DInv or similar software to generate cross-sectional profiles and depth slices as well as 2D/3D models that present the subsurface resistivity distribution. The software produces a subsurface resistivity distribution model after the completion of its inversion process. Using the resistivity model output to determine geological features including the presence of soil layers, bedrock layers, and subsurface water as well as structural elements within the area is called the model interpretation process. By viewing the inverted resistivity model through the visualization tools which include both 2D and 3D displays and contour plots in Res2DInv/ Res3DInv.

## Results

### 2D ERI inversion models

The ERI survey involved sixteen 2D profiles. Fifteen profiles were aligned west–east, and one (Line 16) ran north–south intersecting them. Profile lengths ranged from 70 to 363 m, with penetration depths reaching up to 60 m. Inversion of these profiles, processed over five iterations, resulted in root mean square (RMS) errors between 5.4% and 18.55%, and resistivity values from 1 to 100 Ohm-m.

**Fig. 6:**
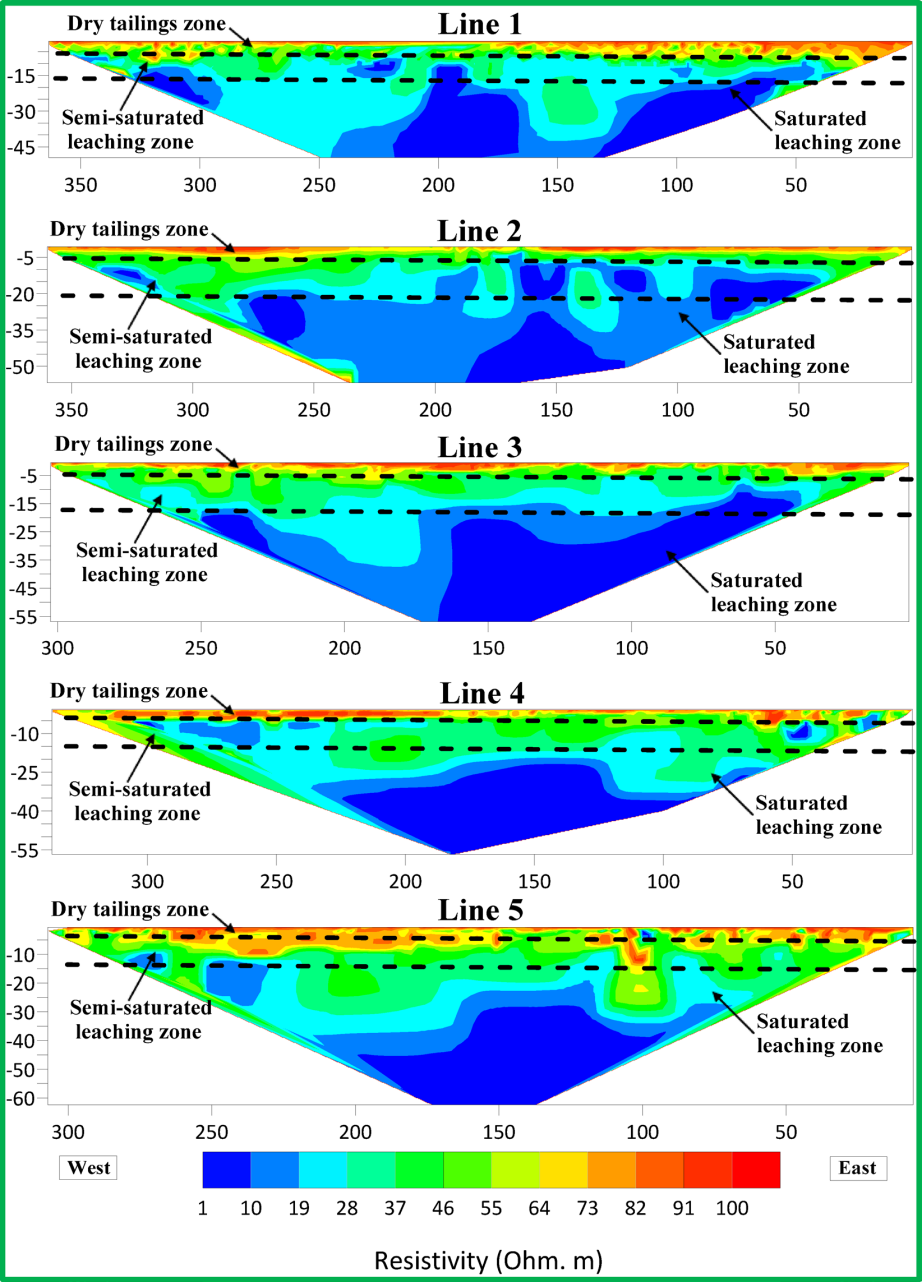
2D inversion models of Lines 1–5.

**Fig. 7:**
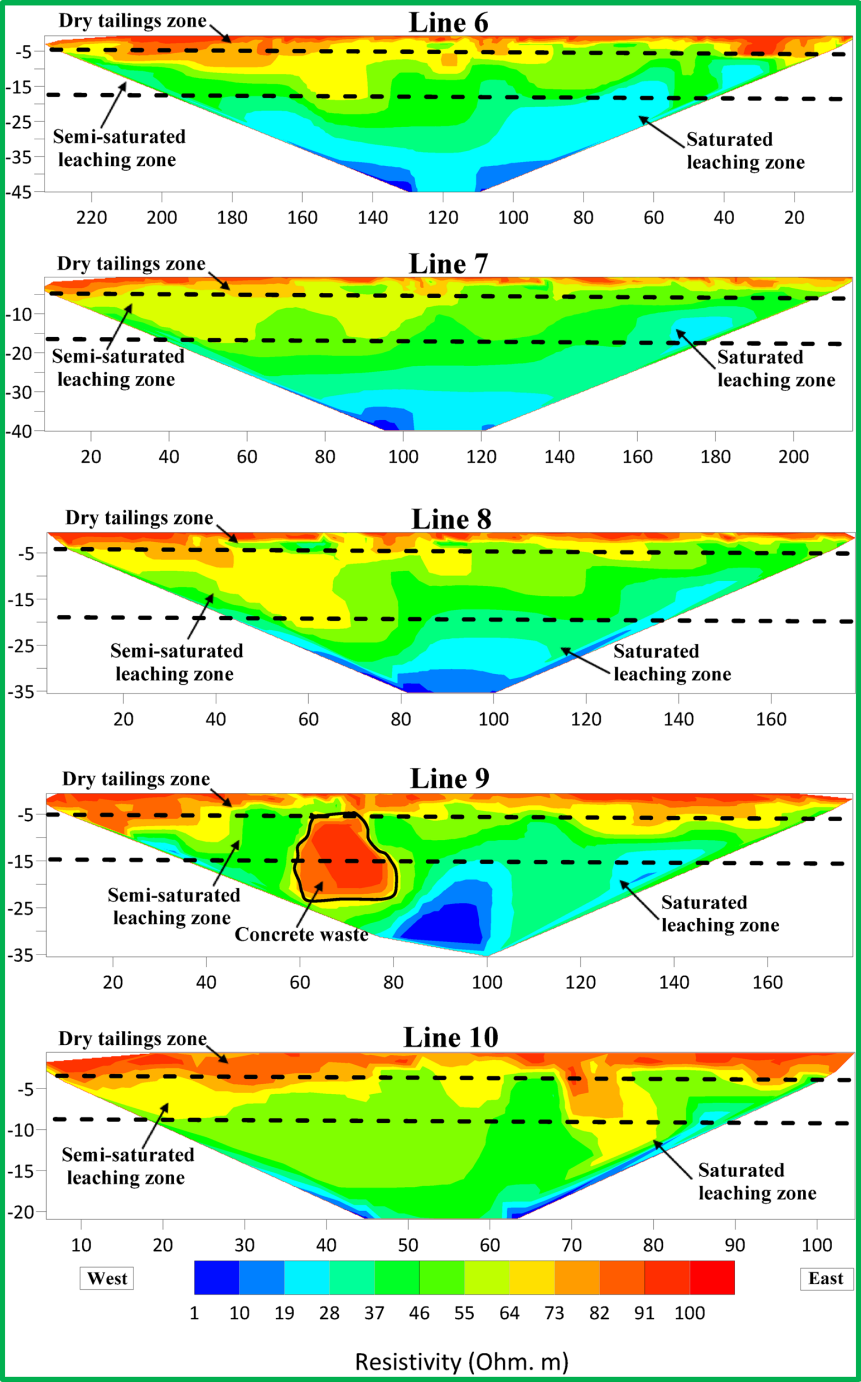
2D inversion models of Lines 6–10.

**Fig. 8:**
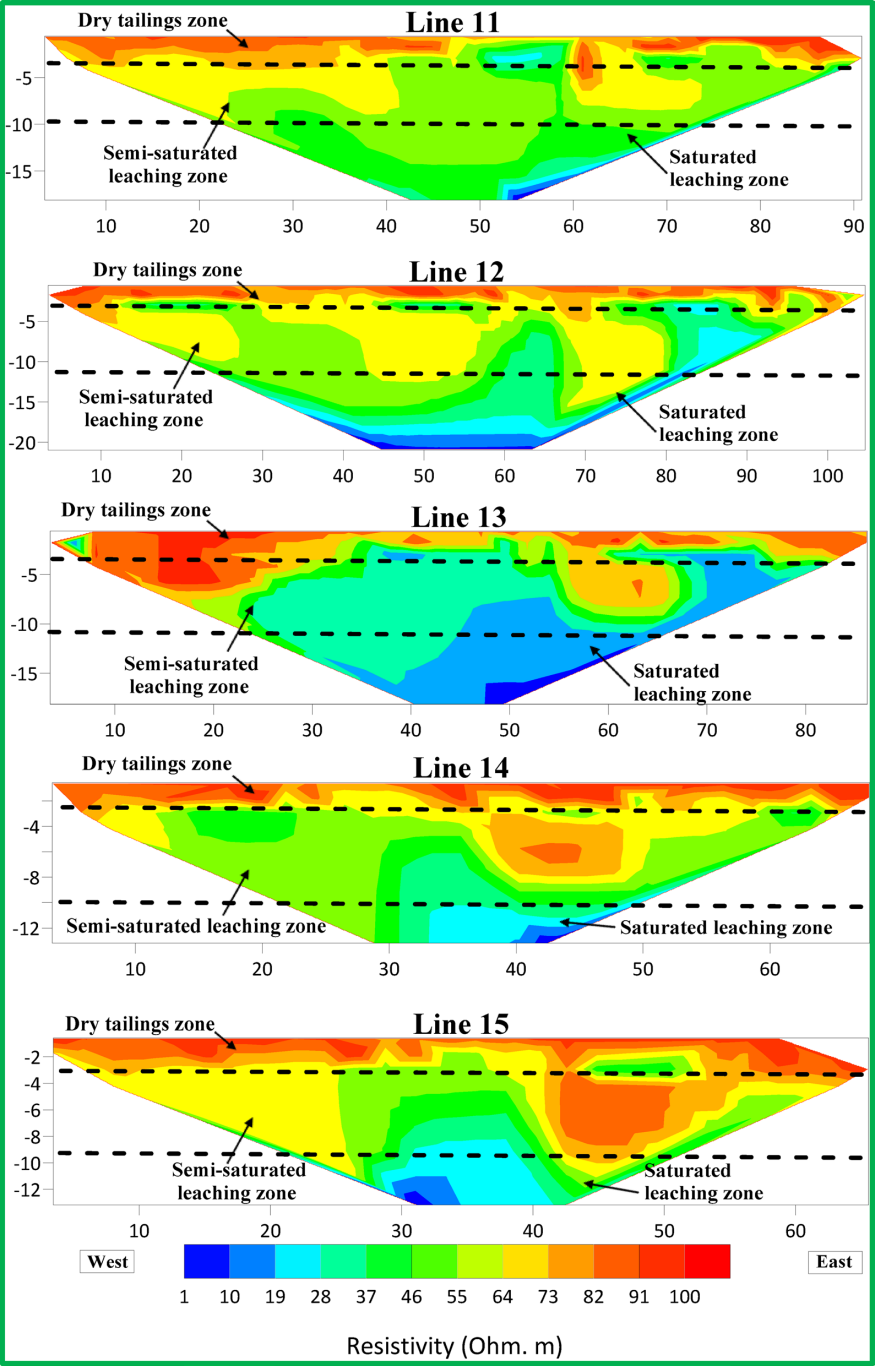
2D inversion models of Lines 11–15.

**Fig. 9:**
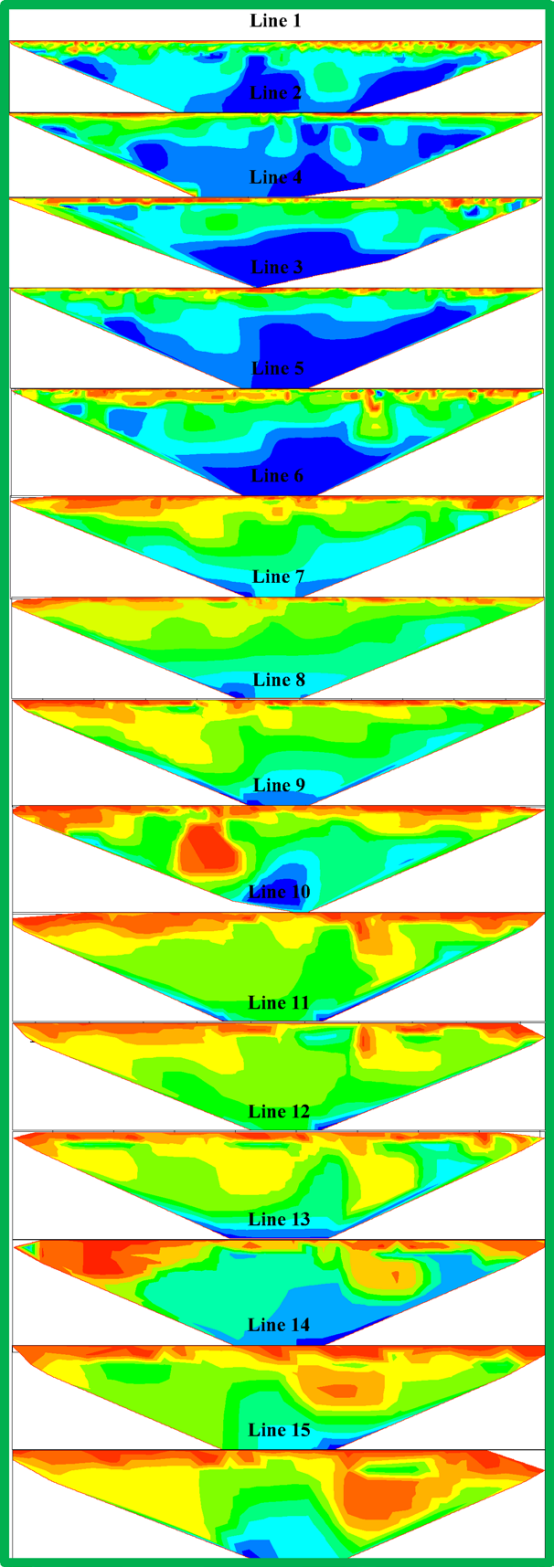
An integrated composite map of the parallel 15 2-D ERI profiles collected across the tailings dump site.

**Fig. 10:**
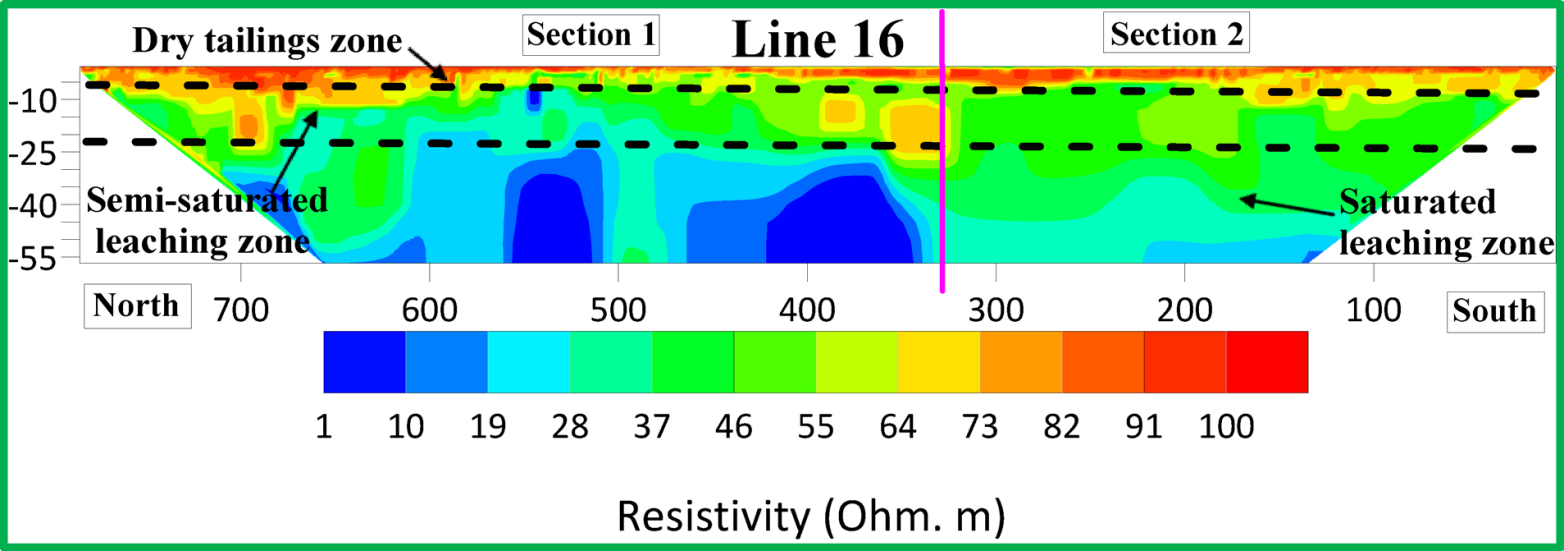
2D inversion models of Line 16.

Figures 6, 7 and 8 show resistivity variations across Lines 1–15. Leachate concentration appears highest in central profiles (Lines 1–5), decreasing gradually in the northern (Lines 6–10) and southern profiles (Lines 11–15). Figure 9 integrates all 15 parallel profiles into a composite resistivity map. Figure 10 presents the cross-sectional profile (Line 16), reinforcing the inferred leachate flow direction.

These results revealed three distinct resistivity zones:

1. Top Zone (High Resistivity, 60–100 Ohm m): Extends 2–3 m deep, indicating dry tailing cover. Anomalies, such as a high-resistivity spherical body in Lines 9 and 10, may reflect filled demolition or concrete waste.
2. Middle Zone (Moderate Resistivity, 30–60 Ohm m): Found 4–15 m below ground level, likely represents partially saturated tailings.
3. Bottom Zone (Low Resistivity, 1–30 Ohm m): Suggests high moisture or leachate accumulation, with irregular boundaries possibly indicating varying permeability and leachate flow behavior.

### 3D ERI inversion models

**Fig. 11:**
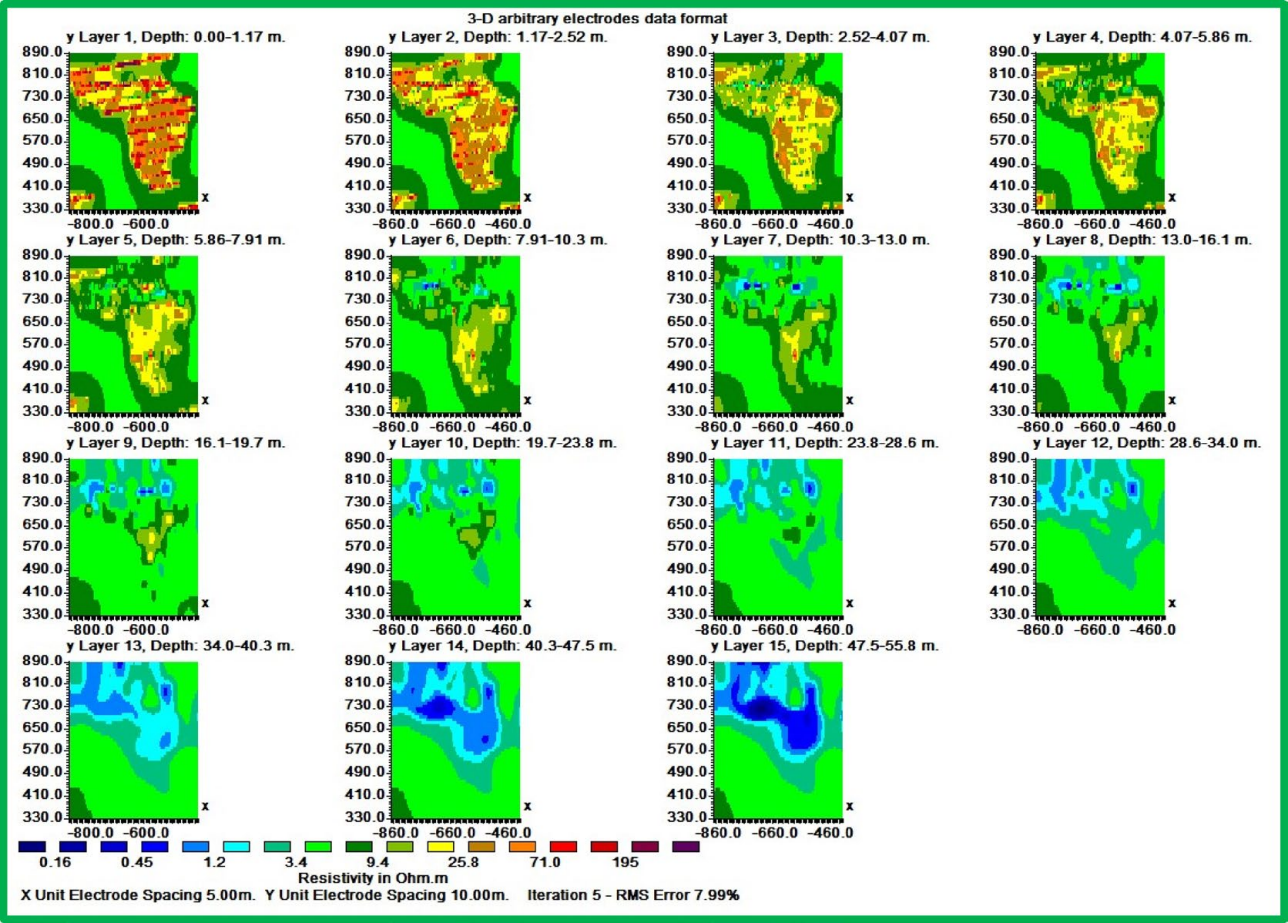
Inverted resistivity model presented as a series of fifteen horizontal depth slices, revealing the spatial distribution of electrical resistivity (in ohm.m) at increasing depths below the surface.

**Fig. 12:**
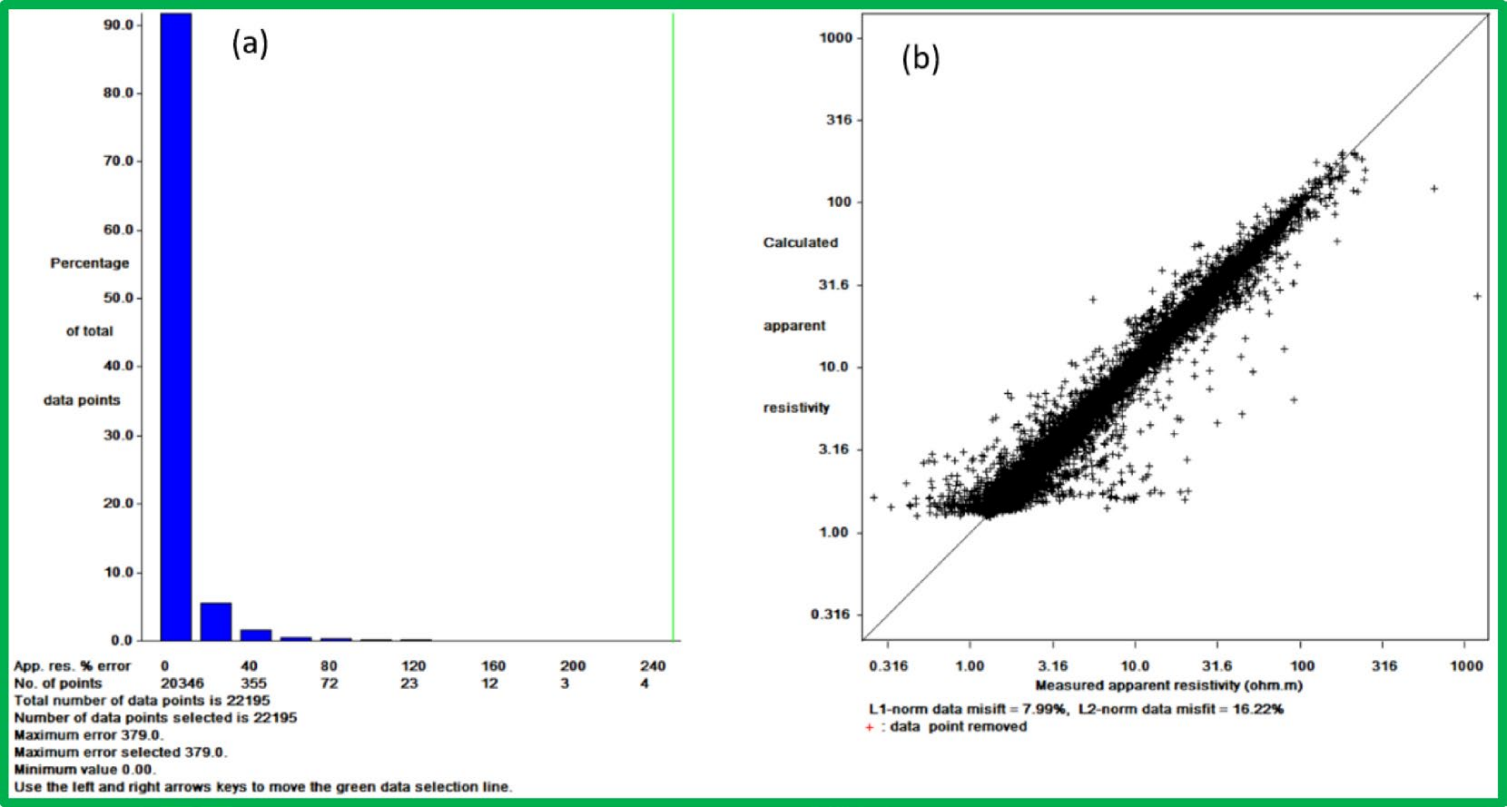
Provides an illustration of the absolute and RMS errors without the exclusion of point outliers for the 3D inversion result. (**a**) a histogram showing the misfit between the calculated and measured apparent resistivity values, (**b**) a scatter plot showing the misfit between the calculated and measured apparent resistivity values.

**Fig. 13:**
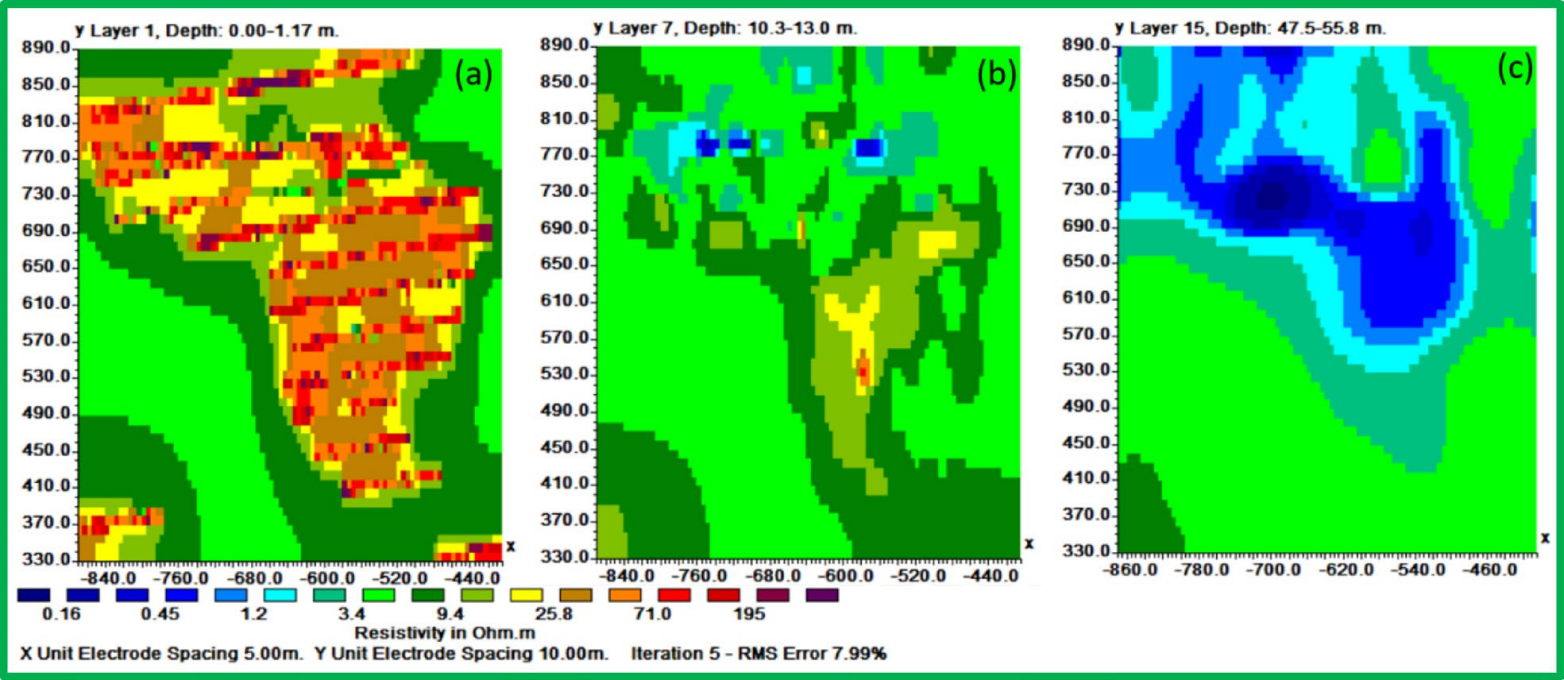
The inverted result shows the electrical resistivity distribution of the three horizontal slices: (**a**) slice 1 at 0.0–1.17 m depth, (**b**) slice 7 at 10.3–13 m depth, and (**b**) slice 15 at 47.5–55.8 m depth.

The 3D inversion, incorporating the 2D datasets, generated 15 horizontal slices visualized in Fig. 11. The model achieved acceptable errors (L1-norm: 7.99%, L2-norm: 16.22%, Fig. 12). Selected slices (Fig. 13) at varying depths show decreasing resistivity with depth, with the lowest values in deeper layers.

**Fig. 14:**
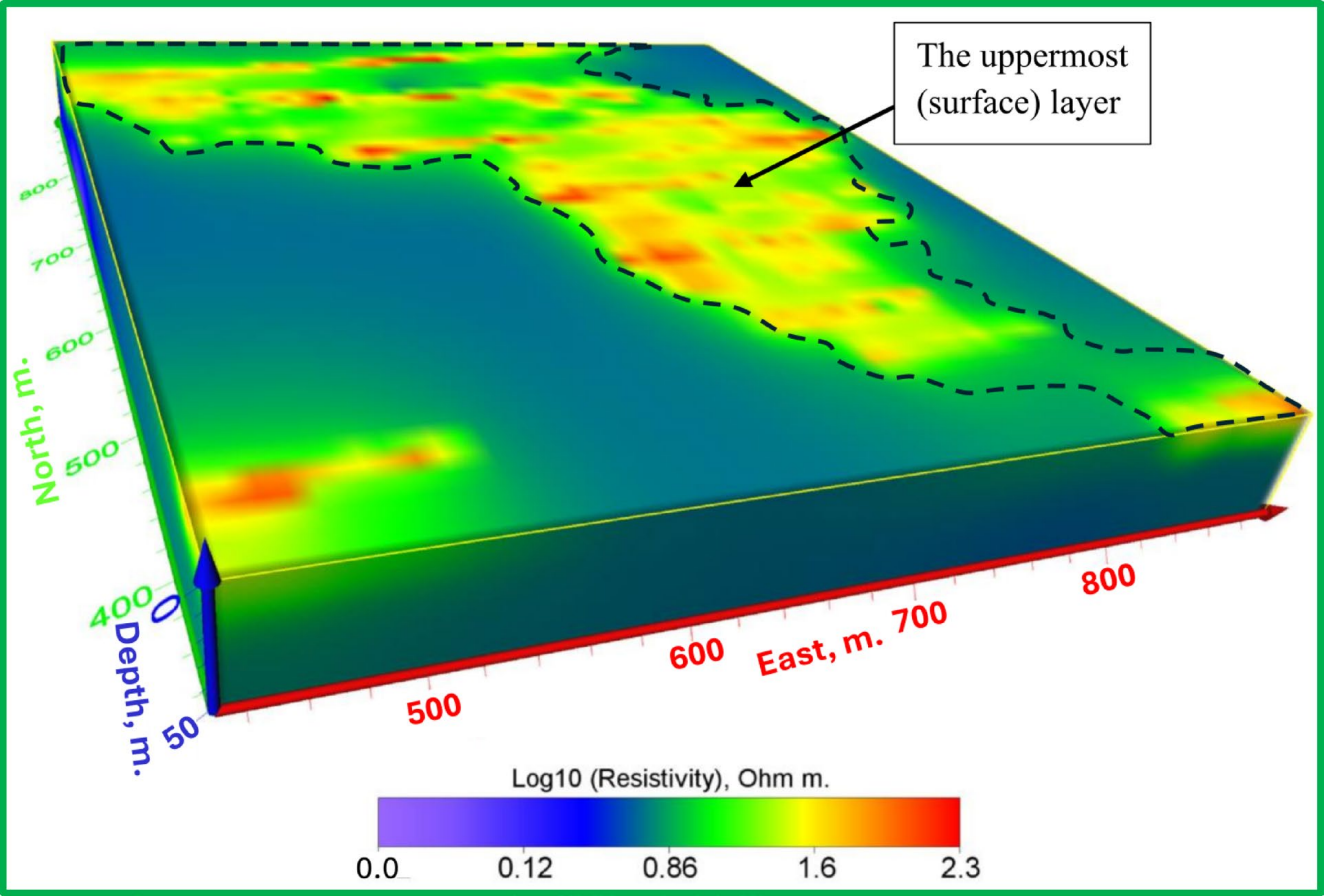
The 3D inverted model shows the electrical resistivity distribution of the uppermost (surface) layer.

**Fig. 15:**
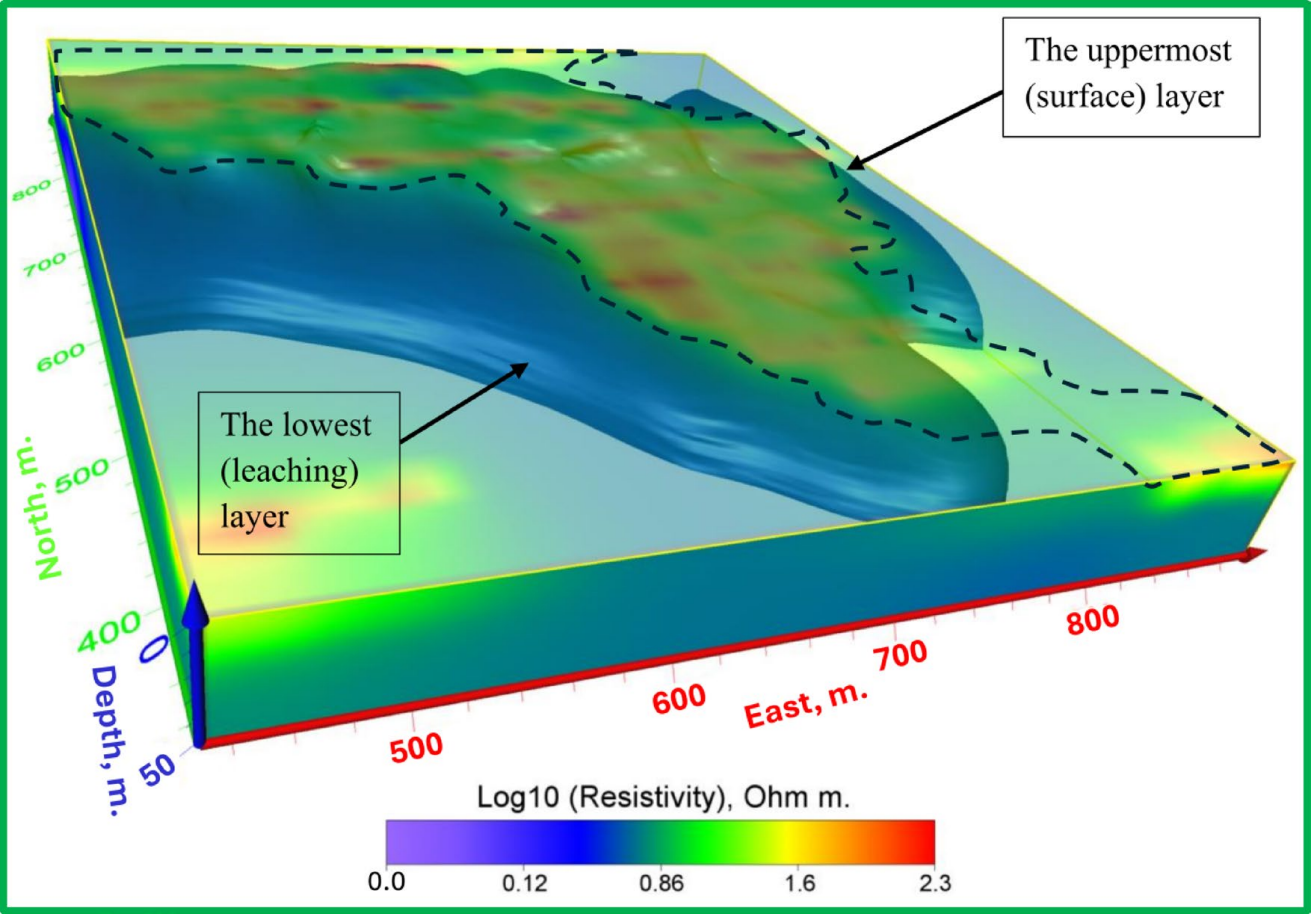
The 3D inverted model shows the electrical resistivity distribution of the uppermost (surface) layer and the lowest (leaching) layer.

**Fig. 16:**
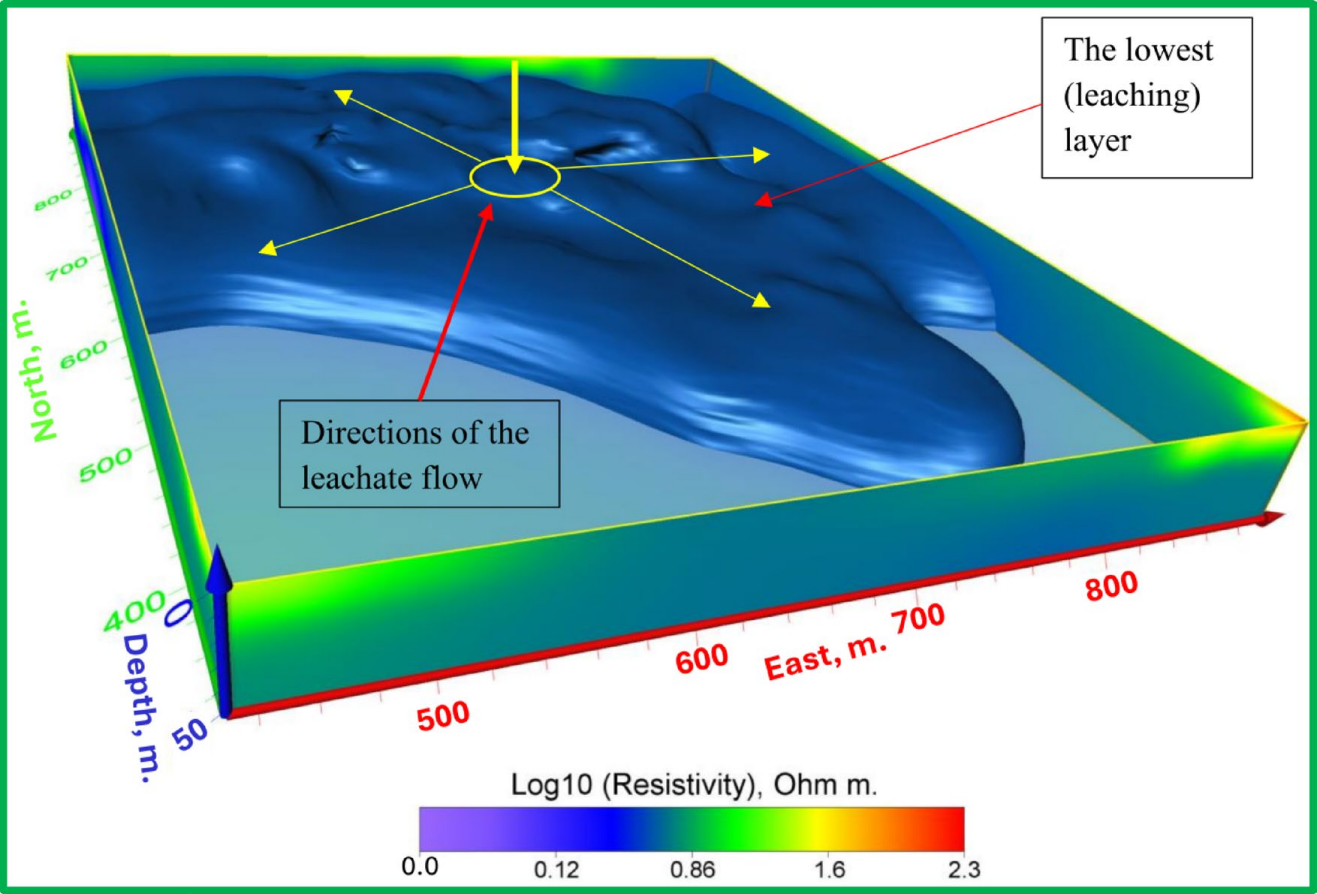
The 3D hydrogeological model shows the location and flow direction of potential leachate.

Figures 14, 15 and 16 display 3D interpretations of resistivity distribution, emphasizing the relationship between surface and deep leaching zones. A hydrogeological iso-surface model (Fig. 16) outlines the potential flow path and spread of leachate.

## Discussion

The discussion of the results needs proper establishment from initial research about the current situation within the study area before delving into the presentation and discussion of the results. The study area consists of historic/old mining tailings that were built up multiple decades since the beginning of mining operations in the region. Expert technicians working at El Mochito Mine provided substantial information about the site through scheduled meetings. The experts’ analysis indicates that the accumulated waste reaches 20 m beyond Earth’s natural surface elevation. The extensive old mining waste layer directly results from unbroken mining activities throughout the area as discussed earlier. These extensive old mining waste accumulations create a major environmental challenge because they can easily pollute regional areas. Our evaluation serves as the first investigation to determine the extent of leachate spread through this waste accumulation area. The leachate should be a critical focus because it shows ability to spread through different areas making adverse effects on the surrounding environment.

In this study, the ERI survey included data acquisition using sixteen 2D profiles among its data collection methods. The fifteen parallel profiles extend from west to east. As shown in Fig. 1 the sixteenth profile crosses through the parallel ones while running in a north–south direction. Each of the parallel ERI profiles ranges in length from 70 to 363 m whereas the cross-profile reaches a measurement of approximately 800 m. A total of sixteen 2D profiles completes this extensive data set for imaging subsurface conditions up to 60 m maximum depth which shows multi-dimensional views of geoelectrical attributes across the study preprimaries hydrogeological characteristics.

The resistivity-based zoning confirms distinct subsurface variations related to leachate saturation and tailings material distribution. The high-resistivity top zone reflects dry conditions, likely engineered tailings covers or construction fill. The middle zone suggests partially saturated conditions, consistent with retained moisture within tailings. The low-resistivity bottom zone is most indicative of conductive, leachate-bearing layers.

The 2D profiles revealed a consistent trend: leachate accumulates centrally and migrates northward, with reduced concentrations toward the south. All resistivity profiles collected at ERI sites showed a clear and uniform zoning pattern after detailed assessment. There are three distinct zones after analyzing resistivity values which serve as categories.

1. The topmost section of the earth known as Top Zone (High Resistivity) extends from 2 to 3 m underground with resistivity readings between 60 to 100 Ohm-meters. The middle section shows characteristics of a dry tailing cover because it consists mainly of solid tailing and waste materials. Most survey lines showed uniformity throughout the lower boundary of this zone although the survey lines 9 and 10 revealed a spherical body below the boundary. The spherical shape indicates a collection point of dry fill materials like concrete and demolition waste which potentially leads to differences in surface cover materials at that location.
2. Between 4 and 15 m below the surface lies the Middle Zone which had moderate resistivity ratings between 30 and 60 Ohm-meters. The intermediate zone functions as a partially saturated area containing tailings.
3. The resistivity values of the lowest zone decrease away from the irregular boundary that separates it from the middle zone. The irregular shape of this boundary demonstrates that the top water table position might not be present since conductive materials seem to leach at different speeds in different locations below the surface. The lowest zone manifests low resistivity ranging from 1 to 30 Ohm-meters. The research data brings essential understanding about the arrangement of subsurface resistivity patterns throughout the depth levels of the tailings dump facility.

To determine leachate flow direction inside this specific area it is essential to connect the surveyed lines. The results shown in Fig. 6 originate from data collected through Lines 1 to 5. The leachate within these lines presents a distinct concentration point with the minimum resistivity values. Evidence indicates leachate accumulation might be dormant in this specific area. The spread of leachate shows a steady decrease from Lines 6 through 10 according to the data presented in Fig. 7. The findings in Lines 11 to 15 of Fig. 8 support the previous observations shown in Figs. 7 and 8. These collective findings show that the leachate flows from the central area of the study site toward the northern region. The leachate concentration levels decrease continuously when traveling towards the south. The study demonstrates that leachate or contaminants move outward from their original central location toward the northern part of the research area. The leachate impact becomes less widespread as the area moves south because the concentration declines in that direction.

Figure 9 presents an integrated composite map of all parallel 15 2-D ERI profiles collected across the El Mochito tailings dump site. This map overlays the individual resistivity sections along their respective survey lines to form a continuous spatial representation of the subsurface resistivity distribution. The accuracy of our interpretation significantly increases through the analysis of data measured at the sixteenth line point as observed in Fig. 10. As shown in the data the leachate dispersion becomes clear to view. The materials seen in Fig. 10 spread farther throughout section “Introduction” extending north from section “Materials and methods” which extends south. The spatial distribution pattern of leachate within the crossing line validates our leachate flow concepts in this study area.

The 3D results validate and enhance these findings, offering a more complete view of vertical and horizontal leachate movement. This is particularly evident in the composite profile map (Fig. 9) and the 3D hydrogeological model (Fig. 16), which together depict both the location and directional flow patterns of leachate.

The Figs. 13, 14 and 15 display an all-encompassing visualization of the examination of leachate from mining tailings dumps through the use of ERI. The uppermost surface zone electrical resistivity distribution appears as a 3D inverted model in Fig. 13. The surface characteristics alongside resistivity values emerge as fundamental information which enables a complete understanding of the subsurface dynamics. The visual depiction in Fig. 14 clearly shows the relationship that exists between the top surface zone and the bottom leaching zone. These two zones produce fundamental interactions according to this depiction which allows researchers to more accurately understand leachate behavior in the study area. Figure 15 offers a 3D iso-surface (hydrogeological) model as the final view to demonstrate both the exact position and flow patterns of potential leachate. The visual presentation demonstrates leachate motion along with its spatial arrangement in the study area which extends our knowledge about possible environmental consequences. The series of Figs. 13 through 15 presented a visual depictions of ERI application for pre-assessing leachate at mining tailings ponds. These analysis tools provide critical data about leachate distribution patterns and movement pathways which supports environmental management programs and pollution remediation decisions.

The 3D models help us tremendously understand how leaching tailings establish their location and behavior patterns throughout the study area. The three-dimensional models deliver vital information regarding leachate spatial movement patterns which enables researchers to validate and enhance their 2D interpretation findings. Technical integration between two-dimensional and three-dimensional modeling produces accurate assessments that define leachate movement patterns and depths which serve environmental management solutions and remedial intervention plans. The resistivity measurements for mining tailings studied by Yurkevich et al. (2017) spanned between 1 to 30 Ohm-meters as reported in^22^.

The study successfully met its initial requirement of mapping pollutant migration routes through location and direction identification but researchers must understand that future all-encompassing research stands on this beginning foundation. Reliable knowledge about complex subsurface dynamics requires combination surveys between geophysics and geochemistry methods.

The 2D and 3D ERI models have produced initial findings about subsurface conditions which reveal possible leachate routes inside the El Mochito mine tailings area. The analysis proves that geophysical surveys including ERI succeed as a reconnaissance approach for handling complex mining tailings management issues at abandoned sites. Additional research alongside advanced study will establish both these preliminary observations and devise effective remediation approaches. The research creates important foundations to develop permanent and environmentally-friendly solutions for mining waste site management.

## Conclusions

This study presents a preliminary evaluation of leachate migration within an old mining tailings disposal site using Electrical Resistivity Imaging (ERI). The site, heavily impacted by decades of industrial mining, contains waste deposits reaching up to 20 m above the natural ground surface. This significant accumulation poses serious environmental risks due to the potential for toxic leachate infiltration into surrounding soils and groundwater. The primary objective was to identify leachate pathways and assess subsurface conditions that influence its movement.

A total of sixteen 2D ERI profiles were collected and analyzed, revealing critical geoelectrical contrasts that allowed for the classification of three distinct subsurface zones:

- The top dry zone, exhibiting high resistivity values (60–100 Ohm m), represents dry tailings with limited moisture content.
- The middle semi-saturated zone showed moderate resistivity (30–60 Ohm m), likely reflecting partially saturated tailings with variable moisture retention.
- The lower conductive zone, with resistivity values ranging from 1–30 Ohm m, is interpreted as a region of leachate accumulation and mineral-rich moisture content.

The analysis of both 2D and integrated inversion models demonstrated that leachate flow is concentrated in the central area and generally migrates northward. The spatial distribution patterns suggest decreasing leachate concentration toward the southern sections, offering essential insights into the site’s hydrogeological behavior. The depth-specific information derived from ERI modeling improves understanding of subsurface contamination pathways and supports informed planning for future environmental remediation.

Despite its valuable contributions, ERI has inherent limitations. The method is indirect and interprets subsurface conditions based on electrical properties, which can be influenced by multiple factors such as moisture content, mineralogy, temperature, and porosity. ERI also suffers from reduced resolution with increasing depth, particularly in heterogeneous or highly conductive environments. Additionally, its effectiveness may be limited in areas with complex subsurface layering or anthropogenic materials, which can introduce inversion artifacts and misinterpretations. Therefore, integrating ERI with complementary techniques—such as geochemical sampling, borehole data, or time-lapse monitoring—is recommended to validate interpretations and enhance diagnostic accuracy.

In conclusion, this investigation establishes a foundational geophysical assessment of leachate movement in a legacy mine tailings site. The results offer critical data for initial environmental risk evaluation and underscore the need for expanded research and multi-method approaches to support the development of effective and sustainable mine waste management strategies.

## Data Availability

All datasets are available upon reasonable request from the corresponding author.
